# Supplementary figures and images for: Structural basis for SARS-CoV-2 neutralizing antibodies with novel binding epitopes
Source: PLoS Biol. 2021 May 7;19(5):e3001209. doi: 10.1371/journal.pbio.3001209 (PMC8133496; doi:10.1371/journal.pbio.3001209)

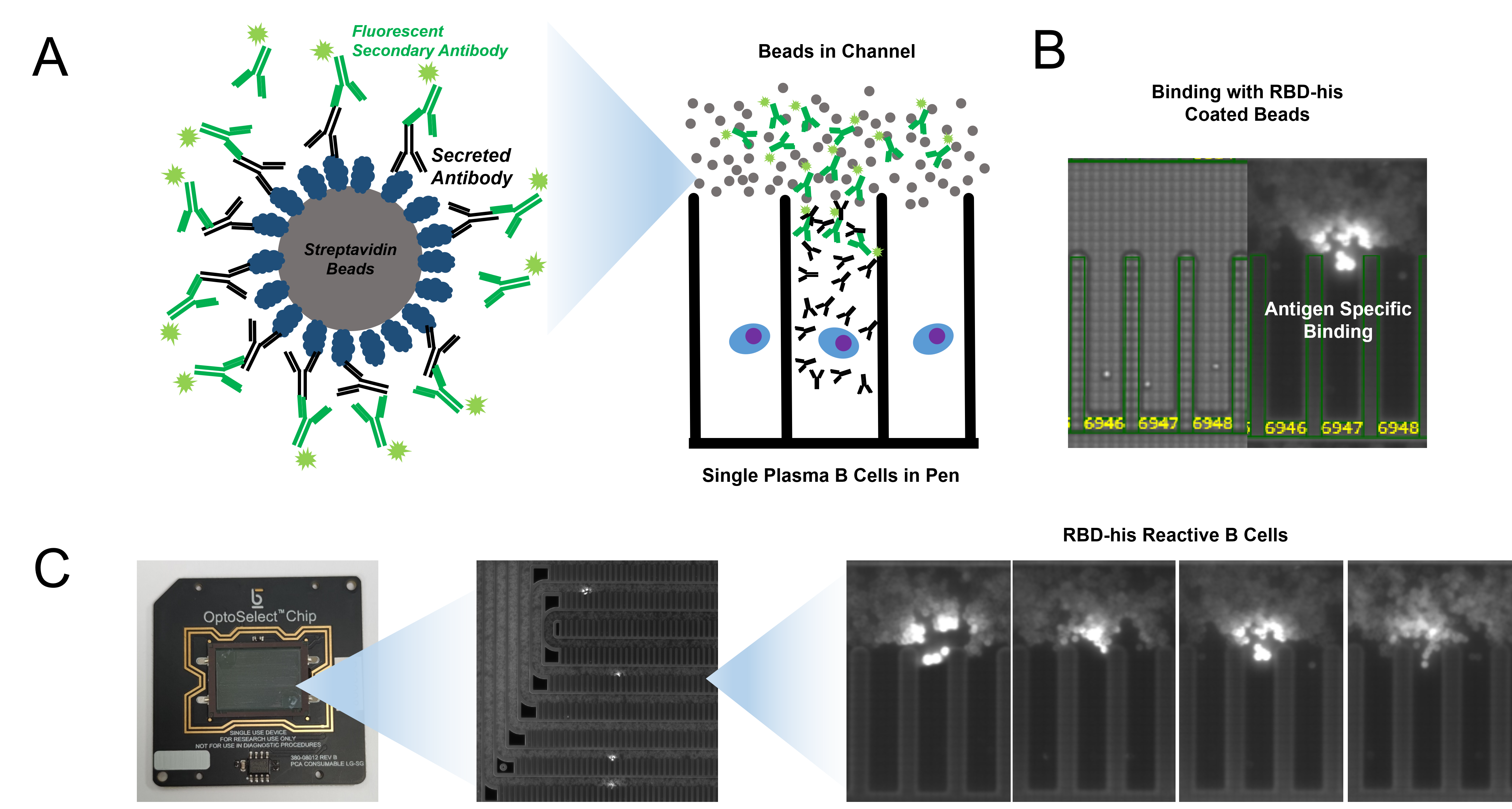

Supplement: S1 Fig — (A) Schematic of in-chip assay on Beacon Optofluidic system to screening for anti-SARS-CoV-2 antibody secreting B cells. Biotinylated antigen (dark blue) was conjugated to a streptavidin-decorated polystyrene bead (dark gray). Single plasma B cells (blue/purple) were loaded into individual NanoPens on the OptoSelect 14K chip in Beacon Optofluidic system, followed by import of a fluorescent secondary antibody-antigen beads mixture. Antibody secreted by plasma B cells binding to antigen was detected by fluorescent anti-rat or anti-mouse IgG secondary antibody (green). (B) Bright field and fluorescent images of NanoPens with single plasma B cells. The plasma B secreting anti-SARS-CoV-2 antibody was identified by increasing fluorescence near the opening of the NanoPen. (C) Representative fluorescent images of an FOV on the OptoSelect 14K chip and zoom in view of NanoPens with positive plasma B cells. FOV, field of view; NAb, neutralizing antibody; SARS-CoV-2, Severe Acute Respiratory Syndrome Coronavirus 2. (TIF) [file pbio.3001209.s001.tif]

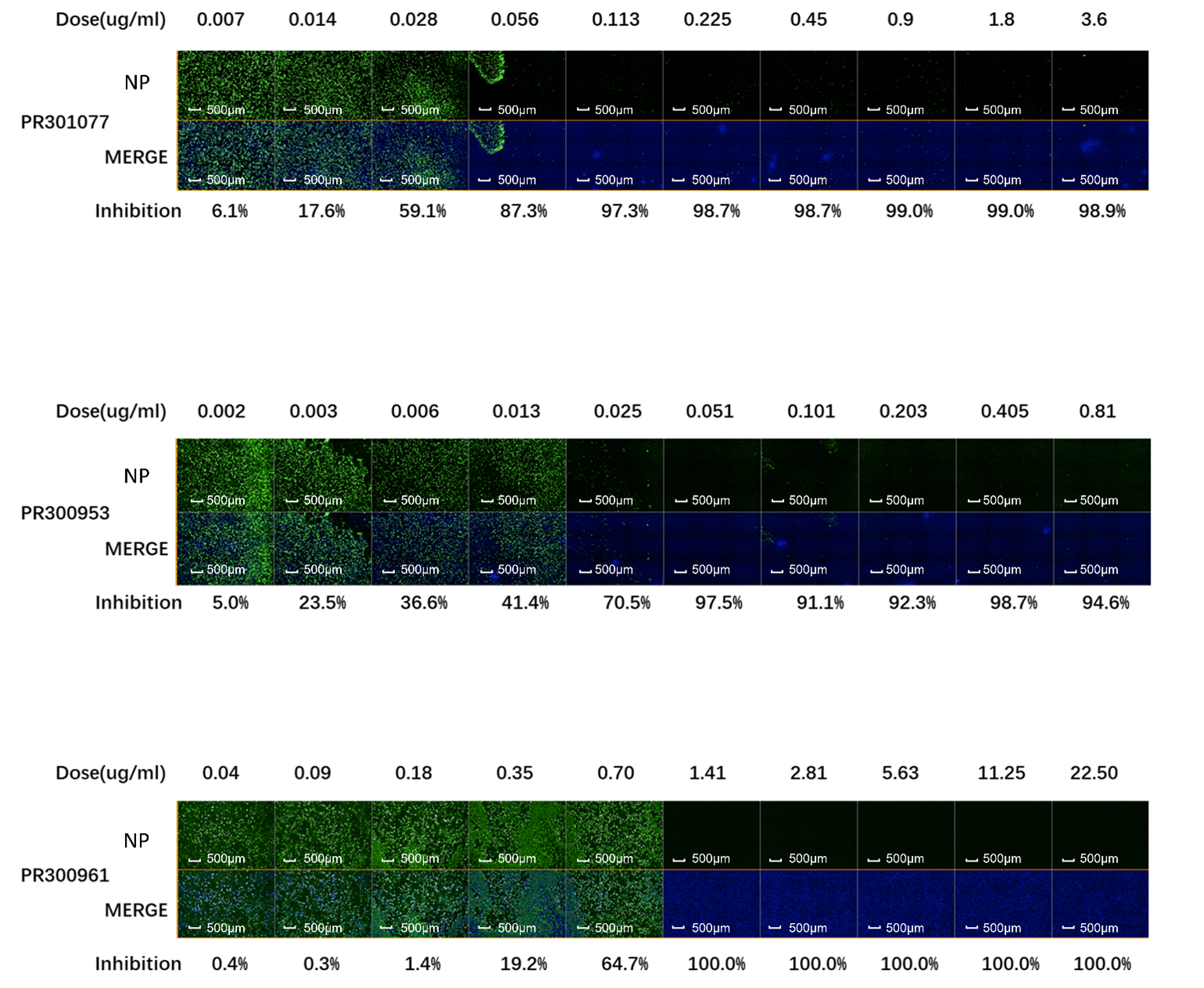

Supplement: S2 Fig — Immunofluorescent staining of SARS-CoV-2 NP for in vitro neutralization assays against live SARS-CoV-2 virus in Vero E6 cells. Green (stained SARS-CoV-2 NP) indicates viral infected cells, and blue (Hoechst 33258) represents cell nuclei. SARS-CoV-2, Severe Acute Respiratory Syndrome Coronavirus 2. (TIF) [file pbio.3001209.s002.tif]

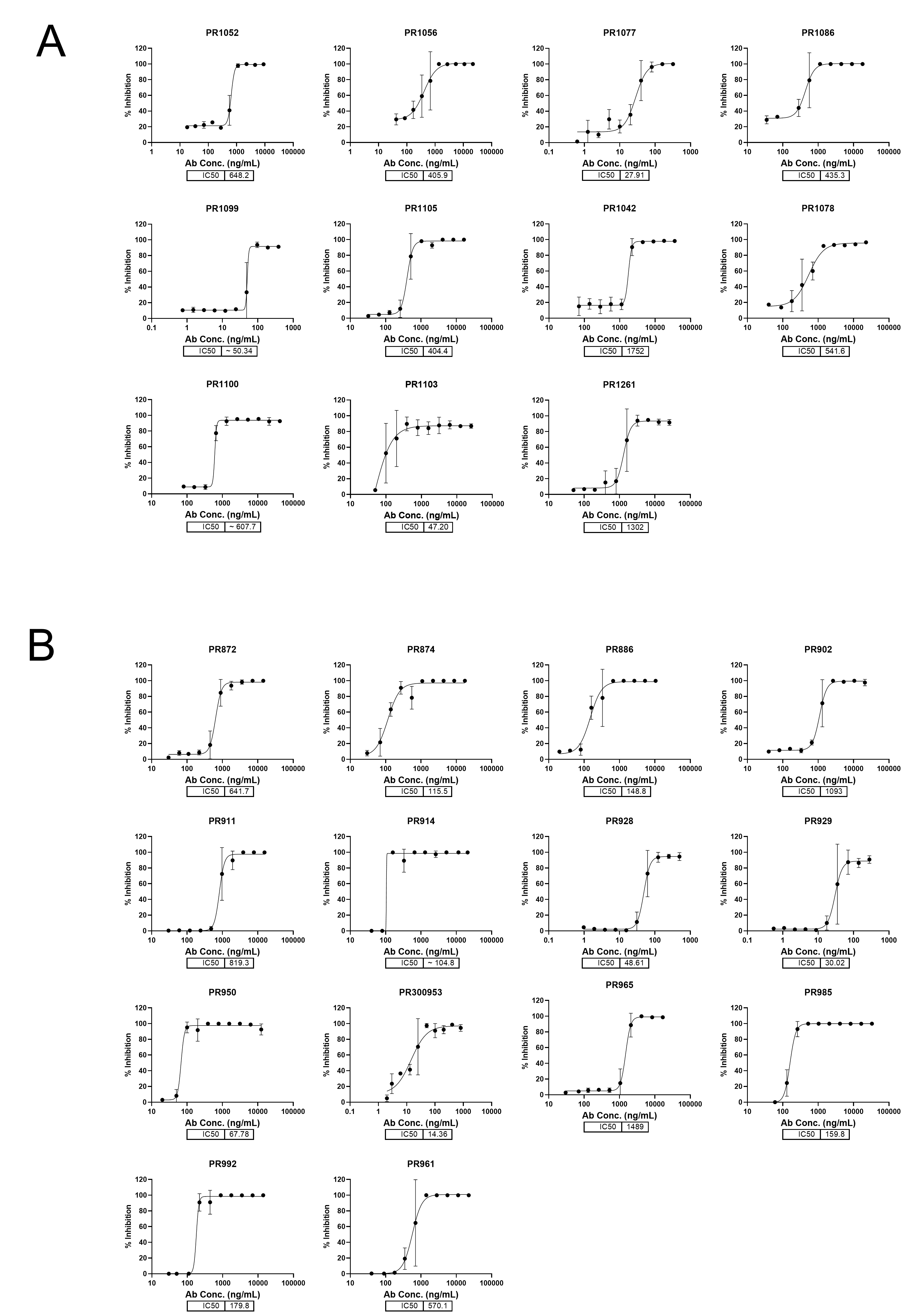

Supplement: S3 Fig — The mixtures of SARS-CoV-2 and serially diluted antibodies were added to Vero E6 cells. After 4 days incubation, IC50 were calculated by fitting the CPE from serially diluted antibody to a sigmoidal dose- response curve. The IC50 were labeled accordingly. (A) SARS-CoV-2 live virus neutralization activity test of 11 mAbs from H2L2 mice. (B) SARS-CoV-2 live virus neutralization activity test of 14 mAbs from BALB/c mice. All the data of this figure can be found in the S11 and S12 Data files. mAb, monoclonal antibody; SARS-CoV-2, Severe Acute Respiratory Syndrome Coronavirus 2. (TIF) [file pbio.3001209.s003.tif]

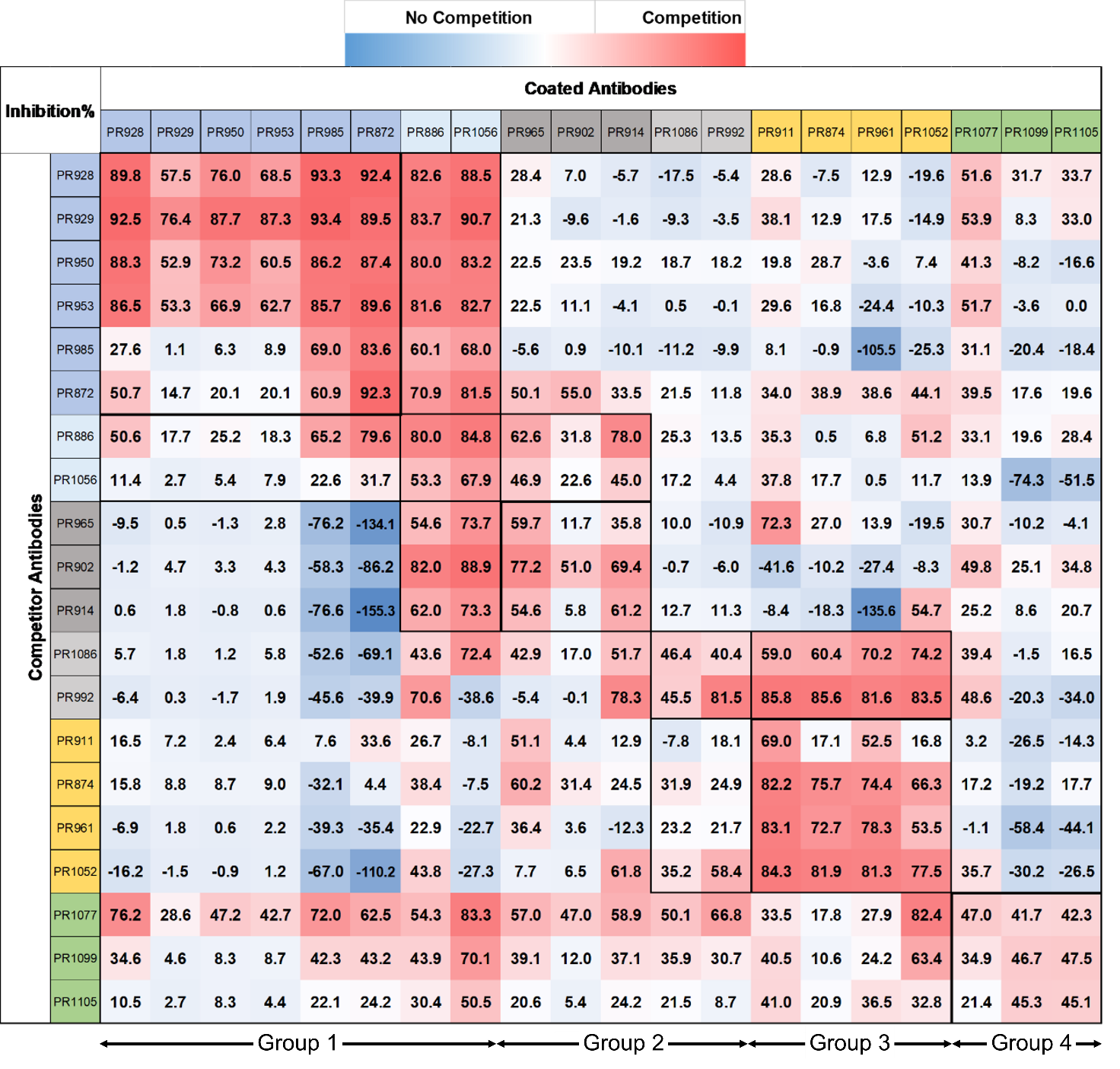

Supplement: S4 Fig — Coated antibody was bound to the substrate in a 96-well high binding plate. Competitor antibody and biotinylated SARS-CoV-2 S1 protein were mixed and incubated for 30 minutes before adding to the plated. After 1-hour incubation, an Alexa 488 streptavidin was used to detect the biotinylated SARS-CoV-2 S1 protein bound to coated antibody. The percentage of inhibition was calculated. Blue indicates no competition, and red indicates competition. The groups 1–4 at the bottom indicates clusters of antibody epitopes defined by the competitive ELISA results. All the data of this figure can be found in the S13 Data file. ELISA, enzyme-linked immunosorbent assay; SARS-CoV-2, Severe Acute Respiratory Syndrome Coronavirus 2. (TIF) [file pbio.3001209.s004.tif]

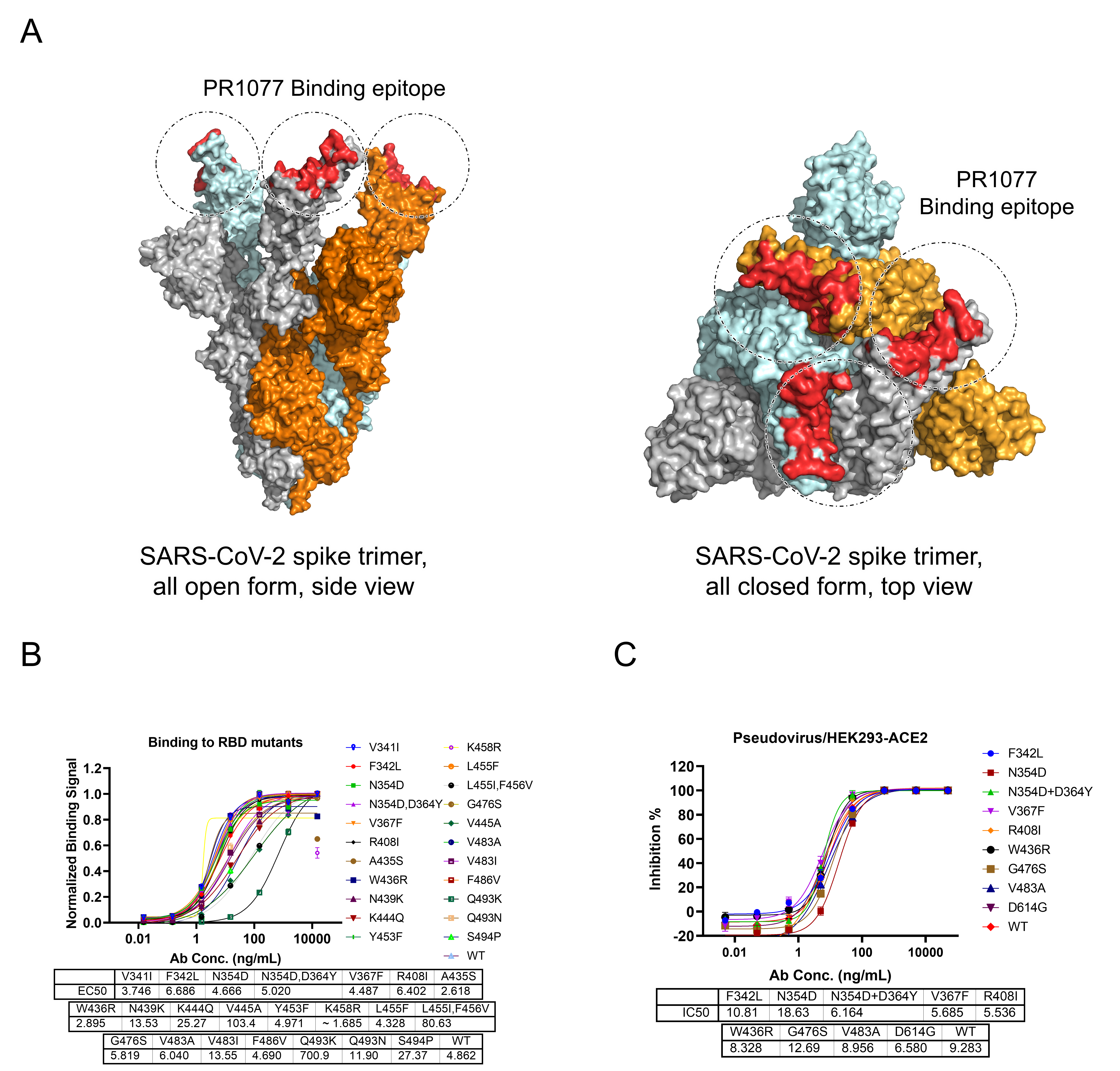

Supplement: S5 Fig — (A) Side or top view of PR1077 binding epitope (colored red) on SARS-CoV-2 spike trimer in “all open form” (superimposed to the spike trimer structure, PDB code: 7CAK) or “all RBD closed state” (superimposed to the spike trimer structure, PDB code: 6ZP0). (B) ELISA binding curves of 22 RBD mutants. PR1077 binds strongly to a wide range of RBD mutants. All the data of this figure can be found in the S14 Data file. (C) PR1077 can effectively neutralize a range of SARS-CoV-2 mutant pseudovirus. All the data of this figure can be found in the S15 Data file. ELISA, enzyme-linked immunosorbent assay; RBD, receptor-binding domain; SARS-CoV-2, Severe Acute Respiratory Syndrome Coronavirus 2. (TIF) [file pbio.3001209.s005.tif]

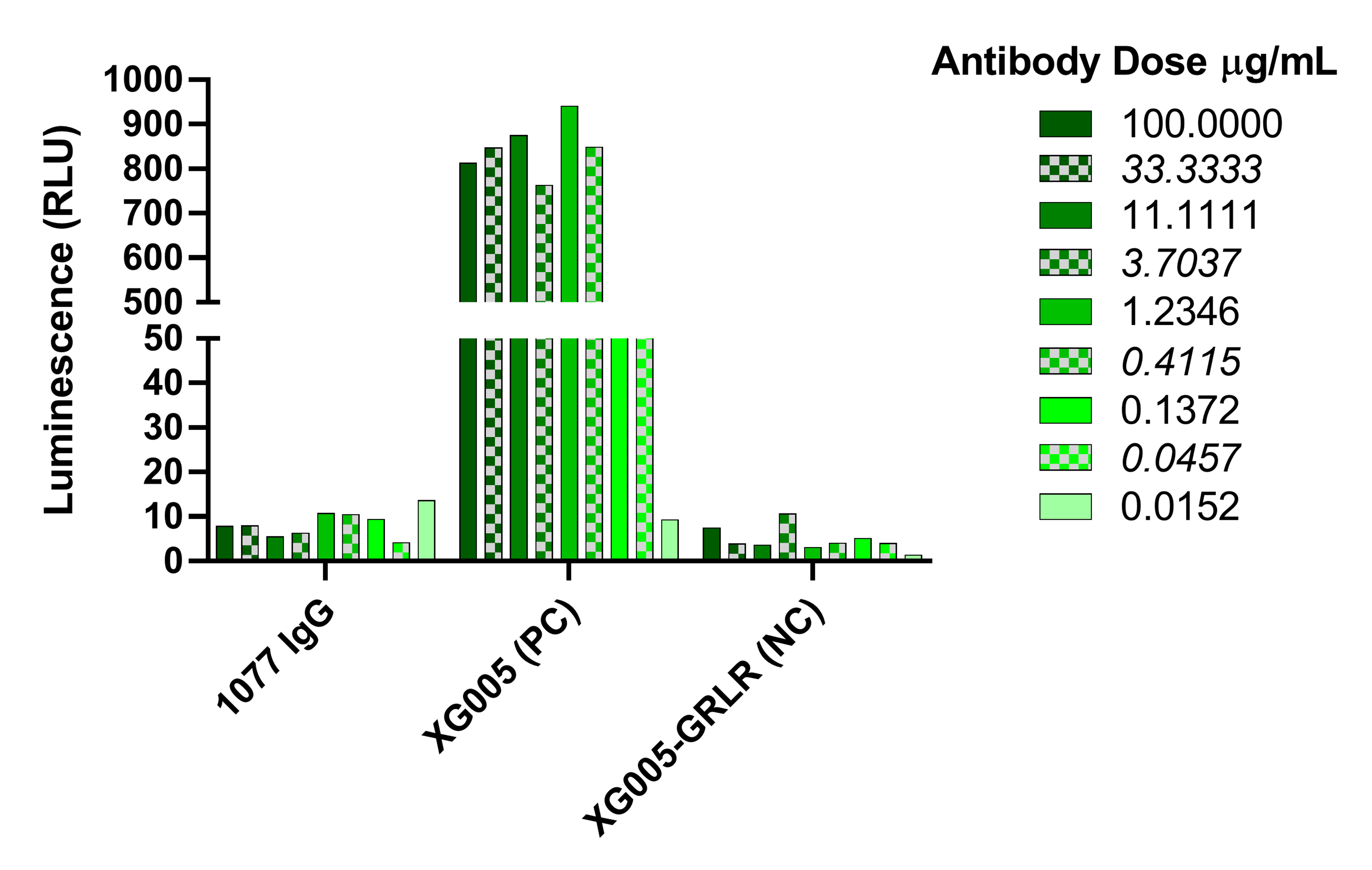

Supplement: S6 Fig — Raji cells as a FcγRII-bearing human B lymphoblast cell line, to study the antibody-dependent viral entry. The antibody-dependent entry of SARS-CoV-2 pseudovirus in the presence of PR1077, XG005, and XG005-GRLR was measured at various antibody concentrations. All the data of this figure can be found in the S16 Data file. ADE, antibody-dependent enhancement; SARS-CoV-2, Severe Acute Respiratory Syndrome Coronavirus 2. (TIF) [file pbio.3001209.s006.tif]

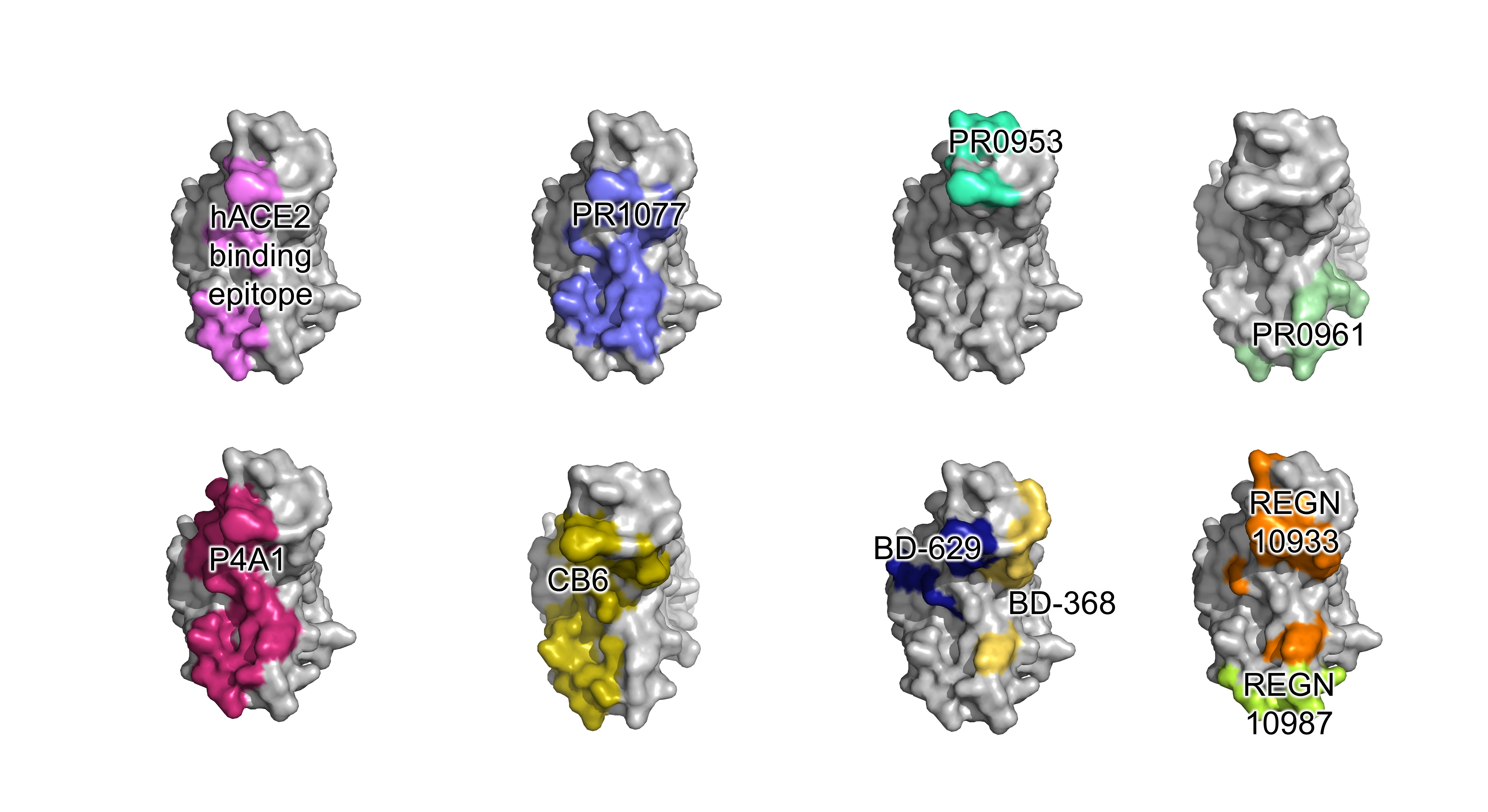

Supplement: S7 Fig — The SARS-CoV-2 RBD is colored in gray and displayed in surface representation. The binding epitope for hACE2 (PDB code: 6M0J), PR1077 (PDB code:7DEO), PR953 (PDB code:7DEU), PR961 (PDB code:7DET), P4A1 (PDB code:7CJF), CB6 (PDB code: 7C01), BD-629 and BD-368 (PDB code: 7CHC), REGN10933, and REGN10987(PDB code: 6XDG) are displayed in different colors. hACE2, human angiotensin-converting enzyme 2; NAb, neutralizing antibody; RBD, receptor-binding domain; SARS-CoV-2, Severe Acute Respiratory Syndrome Coronavirus 2. (TIF) [file pbio.3001209.s007.tif]

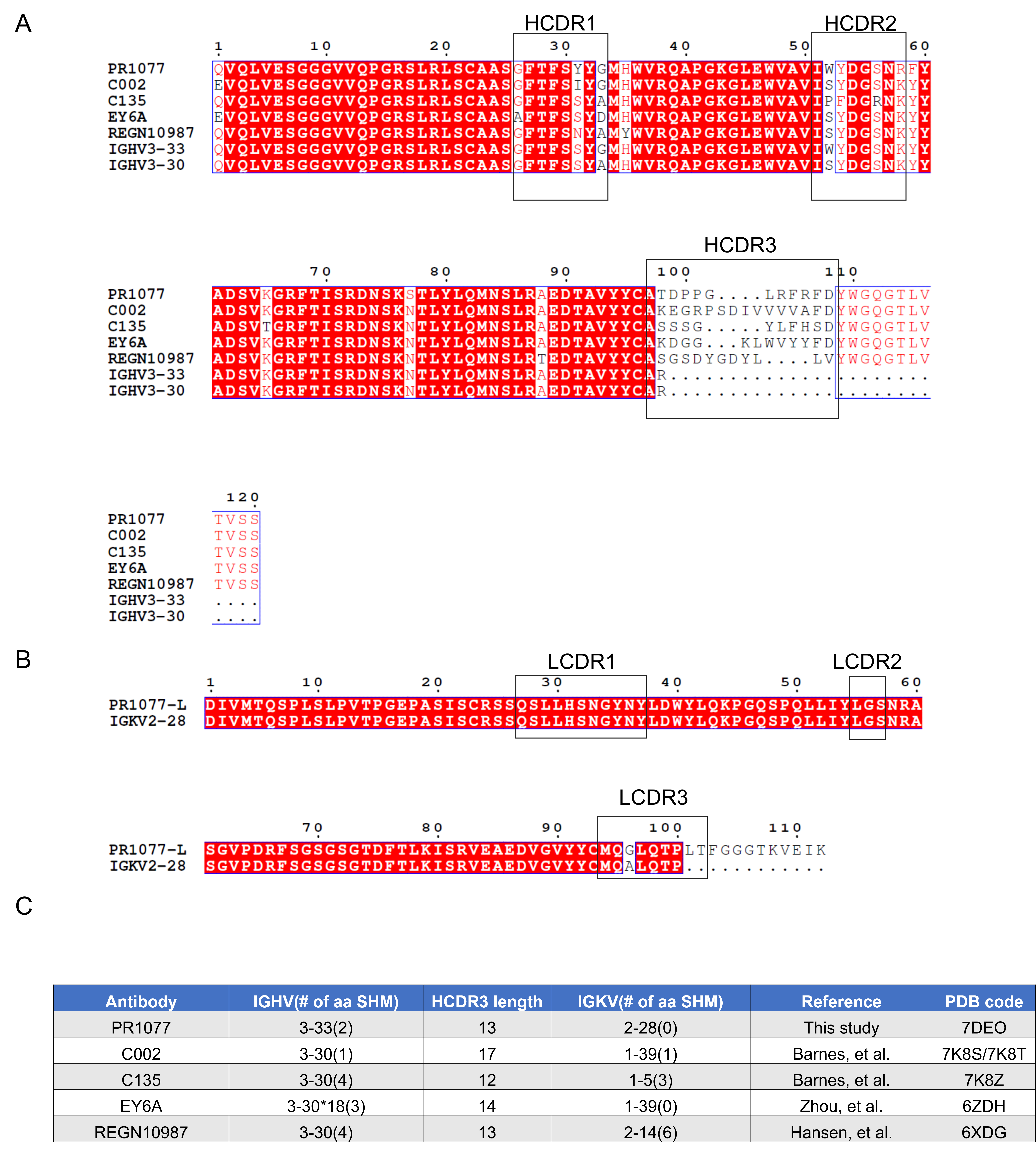

Supplement: S8 Fig — (A) Alignment of the HC variable domain sequence of PR1077 with C002, C135, EY6A, REGE10987 and the germline IGHV3-30, IGHV3-33 sequence. (B) Alignment of the light chain variable domain sequence of PR1077 with the germline IGKV2-28 sequence. (C) Germline usage comparison of the HC and light chain discussed in this paper. HC, heavy chain. (TIF) [file pbio.3001209.s008.tif]

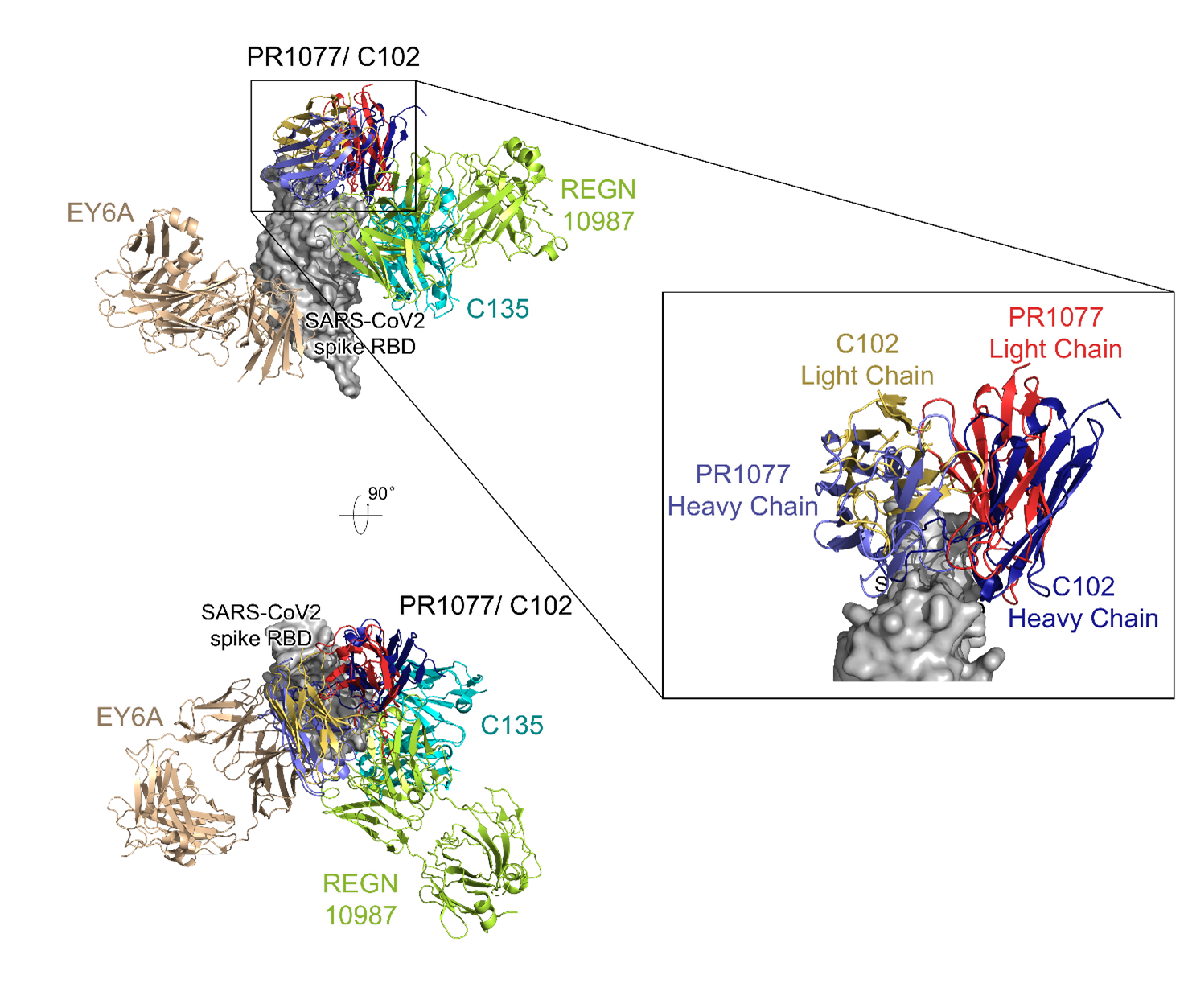

Supplement: S9 Fig — PR1077 (HC: slate blue, light chain: red), C102 (HC: dark blue, light chain: golden yellow), EY6A (yellow orange), C135 (cyan), and REGN10987 (limon green) are superimposed. SARS-CoV-2 spike RBD are shown in surface and colored gray. NAb, neutralizing antibody; RBD, receptor-binding domain. (TIF) [file pbio.3001209.s009.tif]
