# Supplementary material for: Structural basis for SARS-CoV-2 neutralizing antibodies with novel binding epitopes
Source: PLoS Biol. 2021 May 7;19(5):e3001209. doi: 10.1371/journal.pbio.3001209 (PMC8133496; doi:10.1371/journal.pbio.3001209)
Supplement: Attachment — Submitted filename: Response_to_reviewer.docx [file pbio.3001209.s029.docx]

Dear Editor & Reviewers,

The authors greatly appreciate the constructive critiques from the reviewers. The manuscript has been revised according to the reviewers’ comments and we believe this has resulted in improved quality.

Here is our point-to-point response to reviewers’ comments (in blue)

Best,

Yu

REVIEWS:

Reviewer #1: Therapeutic antibodies.

Reviewer #2: Structure-based vaccine design and host immune response-pathogens interactions.

Reviewer #1: Major Points

1.	The authors suggested monoclonal antibody PR1077 binds to the RBD of spike protein in either "open" or "all close state". The manuscript did not provide sufficient experimental data to support this conclusion. As shown in Fig S7, the binding epitope of PR1077 largely overlaps with hACE2 binding region. A CryEM structure of SARS-CoV-2 spike protein trimer has been recently published (Science 2020 367(6483):1260-1263). The authors should specify the critical binding epitopes of PR1077 exposed for surface binding in both "open" or "down" conformations (all close state).

Thank you for this insightful suggestion. We further mapped the epitopes of PR1077 in both “open” or “all closed conformation” spike trimer, the accessible binding surface warrants its binding with all three protomers.

As suggested, we update FigureS5A, and rephrase some text to better illustrate this observation.

2.	Immunofluorescence staining assay was used instead of the classic PRNT assay. In this case, a reliable primer antibody for staining is critical to validate the assay. An in-house prepared and uncharacterized anti-SARS-CoV-2 rabbit serum sample was used to visualize virus infected cells. The authors should use other published neutralizing antibodies to confirm their results (Fig 2F).

Thanks for this suggestion, the authors fully agree with the reviewer that PRNT assay is the classic method to measure IC_50_ value in live virus. Meanwhile, the immunofluorescence labelling assay has also been widely used in SARS-CoV-2 or other viruses. (Wang C, et al. Nat Commun. 2020, May 4;11(1):2251; Zohaib A et al. Emerg Infect Dis. 2020 Jul;26(7):1513-1516 ; Ki Hyun Kim, et al. PLoS Pathog. 2019 Feb 1;15(2):e1007375; Moi M, et al. Methods Mol Biol. 2016;1426:143-52).

Since the initial validation of the assay, we selected several primer antibodies as positive controls, one of them is a published neutralizing antibody, CB6 (reported by Shi, R. et al. Nature. 2020. 584:120-124), this antibody shows similar IC50 value(46ng/ml) comparing with qPCR method (36ng/ml), but since this antibody were not tested parallelly with antibodies reported here, we prefer not to provide these data together with antibodies present in this manuscript.

3.	Has PR1077 been tested against any new SARS-COV-2 variants? This would significantly increase the impact of this story.

Thank you for raising this important concern. Since the initial drafting of the manuscript, we were carefully monitoring RBD and other S protein mutations included in publications and online databases. As we described in Figure.S5B and S5C, PR1077 displayed broad spectrum of binding affinity against all RBD mutants tested (including V367F, R408I, N439K, K444Q, Y453F, Q493N and more), as well as excellent neutralizing activity in pseudovirus assay (including D614G, V367F, R408I and other clinical isolates).

Recently, additional mutant proteins and pseudovirus system are being purified or established, and will be used as soon as they are available. However, as this will be an on-going effort with our continued monitoring of SARS-CoV-2 viral landscape, we would really appreciate the manuscript to be considered for publication ahead of future data generation because of the time-sensitive nature of the subject and potential benefit to the scientific community.

Minor points:

1. Several COVID-19 vaccines have been already approved. The statement "Currently, there is no approved vaccines or therapeutics against COVID-19" in the abstract needs to be revised.

Thank you for this suggestion, the sentence has been corrected accordingly.

2. A statistical test needs to be added in Fig 5B and C for comparison between the PR1077 treated group and PBS control.

Thank you for this suggestion, we updated Fig 5B and C accordingly.

3. More discussion of PR961 neutralization mechanism could be added. PR961 appeared to be a non-receptor blocking neutralizing antibody, which is unique as compared to other published neutralizing antibodies.

Thank you for this suggestion, we added more discussion on this antibody accordingly.

Reviewer #2: Fu et al. reported the use of a transgenic mouse platform to generate antibodies against the RBD of the SARS-CoV-2 spike protein. The authors identified 25 potently neutralizing RBD-specific antibodies and determined the structure of three antibodies with novel epitopes on the RBD. They further showed the prophylactic or therapeutic efficacy of the PR1077 antibody against SARS-CoV-2 infection. In addition, the binding and neutralization ability of PR1077 against numerous emerging RBD mutants was demonstrated. I recommend acceptance with minor revisions.

Minor revisions:

1)	In paragraph 3 of the "Selection and characterization of the purified antibodies" section, the authors claimed that the VH3-33 germline gene usage is among the most frequently used but do not include a citation. Recent reviews do not show this germline gene among the most used but do show the similar VH3-30 gene among the top 5-6. The similarity of the germline genes was later mentioned in the Discussion section when the comparison to published VH3-30 antibody structures was discussed.

Thank you for this comment, germline VH3-33 and VH3-30 share high primary sequence similarities, we rephrase some text and update references accordingly.

2)	Figure 1D is never cited in the manuscript.

Thank you for this comment, we apologize for this carelessness, we have updated the description accordingly.
