# Supplementary material for: Structural basis for SARS-CoV-2 neutralizing antibodies with novel binding epitopes
Source: PLoS Biol. 2021 May 7;19(5):e3001209. doi: 10.1371/journal.pbio.3001209 (PMC8133496; doi:10.1371/journal.pbio.3001209)
Supplement: Attachment — Submitted filename: Response to Reviewer.docx [file pbio.3001209.s030.docx]

Dear Editor & Reviewers,

The authors greatly appreciate the constructive critiques from the editor and reviewers. The manuscript has been revised according to the reviewers’ comments and we believe this has resulted in improved quality.

Here is our point-to-point response to reviewers’ comments (in blue)

Sincerely,

Yu

You may be aware of the PLOS Data Policy, which requires that all data be made available without restriction: http://journals.plos.org/plosbiology/s/data-availability. For more information, please also see this editorial: http://dx.doi.org/10.1371/journal.pbio.1001797

Response: confirmed

Note that we do not require all raw data. Rather, we ask that all individual quantitative observations that underlie the data summarized in the figures and results of your paper be made available in one of the following forms:

1) Supplementary files (e.g., excel). Please ensure that all data files are uploaded as 'Supporting Information' and are invariably referred to (in the manuscript, figure legends, and the Description field when uploading your files) using the following format verbatim: S1 Data, S2 Data, etc. Multiple panels of a single or even several figures can be included as multiple sheets in one excel file that is saved using exactly the following convention: S1_Data.xlsx (using an underscore).

2) Deposition in a publicly available repository. Please also provide the accession code or a reviewer link so that we may view your data before publication. 

Regardless of the method selected, please ensure that you provide the individual numerical values that underlie the summary data displayed in the following figure panels as they are essential for readers to assess your analysis and to reproduce it: Figure 1B, 1C, 2A, 2B, 2C, 2D, 2E, 2F, 5B, 5C, S3A, S3B, S4, S5B, S5C, and S6.

Response: The individual numerical values of Figure 1B, 1C, 2A, 2B, 2C, 2D, 2E, 2F, 5B, 5C, S3A, S3B, S4, S5B, S5C, and S6 have been upload as supplementary files.

Please also provide size bars for the microscopy pictures in figure S2.

Response: We update figure S2 accordingly.

NOTE: the numerical data provided should include all replicates AND the way in which the plotted mean and errors were derived (it should not present only the mean/average values).

**Please also ensure that figure legends in your manuscript include information on where the underlying data can be found, and ensure your supplemental data file/s has a legend.**

Response: Confirmed.

Please ensure that your Data Statement in the submission system accurately describes where your data can be found.

Response: Confirmed.

As you address these items, please take this last chance to review your reference list to ensure that it is complete and correct. If you have cited papers that have been retracted, please include the rationale for doing so in the manuscript text, or remove these references and replace them with relevant current references. Any changes to the reference list should be mentioned in the cover letter that accompanies your revised manuscript.

Response: Confirmed. No change has been made to the reference list.


------------------------------------------------------------------------
Reviewer remarks:

Reviewer #1: The revised version addressed reviewer's concerns. It is recommended for acceptance.

Response: We thank the reviewer for his/her constructive comments.
